# CCM1 and CCM2 variants in patients with cerebral cavernous malformation in an ethnically Chinese population in Taiwan

**DOI:** 10.1038/s41598-019-48448-y

**Published:** 2019-08-27

**Authors:** Chun-Wei Chang, Peng-Wei Hsu, Kuo-Chen Wei, Chia-Wen Chang, Hon-Chung Fung, Mo-Song Hsih, Wen-Chuin Hsu, Long-Sun Ro, Chen-Nen Chang, Jiun-Jie Wang, Yih-Ru Wu, Sien-Tsong Chen

**Affiliations:** 1 Department of Neurology, Chang Gung Memorial Hospital - Linkou Medical Center, Taoyuan, Taiwan; 2 Department of Neurosurgery, Chang Gung Memorial Hospital - Linkou Medical Center, Taoyuan, Taiwan; 3 College of Medicine, Chang Gung University, Taoyuan, Taiwan; 4 Department of Neurosurgery, Xiamen Chang Gung Memorial Hospital, Xiamen, China; 5 Department of Medical Imaging and Radiological Sciences, Chang Gung University, Taoyuan, Taiwan; 6 Healthy Ageing Research Center, Chang Gung University, Taoyuan, Taiwan

**Keywords:** Cerebrovascular disorders, Clinical genetics

## Abstract

Cerebral cavernous malformation (CCM) is a vascular malformation characterized by clustered enlarged capillary-like channels in the central nervous system. The genes harboring variants in patients with CCM include *CCM1*/Krev interaction trapped-1, *CCM2*/MGC4607, and *CCM3*/programmed cell death protein 10. We aimed to identify pathogenic variants in an ethnic Chinese population in Taiwan. We recruited 95 patients with multiple CCMs or a single lesion with a relevant family history. Sanger sequencing was performed for 41 patients. Variants were identified using sequence alignment tools, and the clinical significance of these variants was determined using American College of Medical Genetics and Genomics standards and guidelines. Several pathogenic variants were found in six patients, including three unrelated patients and three affected members of one family. Two novel pathogenic variants leading to early truncation comprised a deletion variant in exon 18 of *CCM1* (c.1846delA; p.Glu617LysfsTer44) and an insertion variant in exon 4 of *CCM2* (c.401_402insGCCC; p.Ile136AlafsTer4). One novel pathogenic splice site variant was c.485 + 1G > C at the beginning of intron 8 of *CCM1*. In this study, we identified novel variants related to CCM in an ethnically Chinese population in Taiwan.

## Introduction

Cerebral cavernous malformation (CCM, OMIM 116860) is a vascular malformation characterized by clustered enlarged capillary-like channels and the absence of the intervening neural tissue. The prevalence of CCM in the general population is estimated to be 0.5%^1^. CCM can be sporadic or familial, and familial cases usually have multiple lesions^2^. The symptoms of CCM include hemorrhage-related focal neurological deficits, seizure, and headache; however, patients with CCM are usually asymptomatic^1^. In familial individuals, three related genes, namely *CCM1* (Krev interaction trapped-1, OMIM 604214), *CCM2* (MGC4607, OMIM 607929), and *CCM3* (programmed cell death protein 10, OMIM 609118), have been reported^3–5^. The inheritance is autosomal dominant with incomplete clinical and neuroradiological penetrance^2^. Multiple pathogenic variants have been reported in these genes, and several studies have been conducted in ethnically Chinese populations^6–19^, with only one case reported in Taiwan^20^. Therefore, we retrospectively collected clinical and magnetic resonance imaging (MRI) data of patients who had received a diagnosis of CCM on the basis of brain MRI findings from 1998 to 2006 in a tertiary medical center. Thereafter, we performed DNA analysis to identify variants in patients with multiple lesions or with relevant family histories. We aimed to establish an epidemiological and clinical data bank and identify CCM pathological variants in an ethnic Chinese population in Taiwan.

## Results

### Basic demographic profile and clinical presentation

We recruited 95 patients with multiple CCMs or a single CCM with a relevant family history (Table 1). Of the 95 patients, 15 (15.8%) had a relevant family history and the remaining 80 (84.2%) had a sporadic onset. 92 patients (96.8%) had multiple cerebral lesions and only 3 (3.2%) had a single lesion in their brain. The most common initial presentation was focal neurological signs (54 patients, 56.8%), including weakness, numbness, diplopia, and dysarthria, followed by seizure (28 patients, 29.5%; 3.2% with concurrent focal neurological signs) and headache (21 patients, 22.1%; 13.7% with concurrent focal neurological signs or seizure); however, only eight patients (8.4%) were asymptomatic.

**Table 1:** Demographic data of the recruited patients.

			Patient Number	Percentage
Onset Age (years)		38.97 ± 19.9		
Sex (n)	Male		49	51.6
	Female		46	48.4
Family history (n)	Yes		15^a^	15.8
	No		80	84.2
Underlying disease (n)	Hypertension		25	26.3
	Heart disease		2	2.1
	Diabetes mellitus		7	7.4
	Coagulopathy		0	0.0
	Malignancy		9	9.5
Lesion number (n)	Multiple		92	96.8
	Single		3	3.2
Lesion site (n)	Supra-tentorial		46	48.4
	Extra-cranial		2	2.1
	Infra-tentorial		26	27.4
	Concurrent		23	24.2
Initial presentation (n)	Focal neurological signs		54	56.8
	Weakness		18	18.9
	Vertigo		10	10.5
	Diplopia		10	10.5
	Numbness		8	8.4
	Tinnitus		2	2.1
	Dysarthria		2	2.1
	Gait disturbance		2	2.1
	Facial palsy		1	1.1
	Visual field defect		1	1.1
	Seizure		28	29.5
	Alone		25	26.3
	With focal signs		3	3.2
	Headache		21	22.1
	Alone		8	8.4
	With focal signs		10	10.5
	With seizure		3	3.2
	Incidental finding		8	8.4
Intervention (n)	Surgical removal		30	31.6
	Radiotherapy		38	40.0
Gene Study (n)	Done		41	43.2
	Not done		54	56.8

^a^12 unrelated patients and 3 affected members of one family.

In addition, 46 (48.4%), 26 (27.4%) and 23 (24.2%) patients had supratentorial hemangioma, infratentorial lesions, and both supratentorial and infratentorial lesions, respectively. Only two (2.1%) patients had extracranial cavernous hemangioma at the spinal cord. Among the 95 patients, 30 (31.6%) underwent surgical lesion removal and 38 (40.0%) received radiosurgery before this study because of recurrence or critical lesion sites.

### DNA analysis

**Figure 1:**
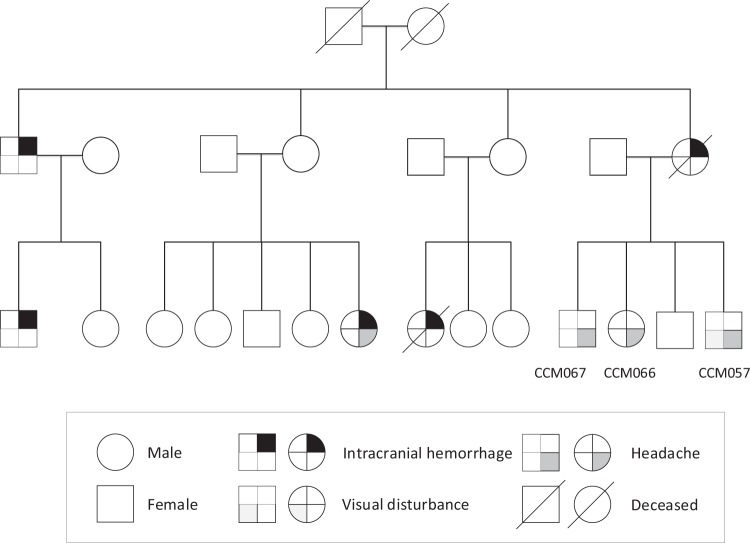
Pedigree of three affected patients with one pathogenic *CCM1* variant from one family. A square represents a male patient, whereas a circle represents a female patient. A line across the symbol indicates the patient is deceased.

Of the 95 patients, we collected blood samples from 41 (43.1%), which comprised 29 patients with unrelated sporadic cases, 9 with unrelated familial cases, and 3 who were affected members of one family. The pedigree of this family is depicted in Fig. 1. We could not obtain blood samples from the remaining 54 patients because of loss to follow-up (47) or patients’ refusal (7). Five patients and one patient had pathogenic *CCM1* and *CCM2* variants, respectively; additionally, one patient possessed a *CCM2* variant of uncertain significance. The clinical manifestations and gene analysis results are listed in Table 2. Among the patients with variants, one (Patient 20) underwent both surgical removal and radiotherapy for hemisphere lesions and another (Patient 54) received only radiotherapy because of symptomatic CCMs. One patient (Patient 20) experienced another episode of paraplegia, and a follow-up MRI study revealed a cavernous hemangioma in the spinal cord.

**Table 2:** Detailed information of patients with the variants of *CCM1*, *CCM2*, and *CCM3*.

No.	Sex	Lesions number	Symptoms	Lesions location	Family	Gene	Site	Detail	Predictors score
20	Female	>200	Weakness	Cerebrum Spine	No	*CCM1*	Exon 18	1. c.1844G > C^c^ 2. c.1846delA^b,c^	P: 0.97, M: 0.99 S: 0.76 M: frameshift (1.0) S: frameshift (0.85)
40	Female	18	Seizure	Cerebrum	No	*CCM1*	Intron 8	c.485 + 1G > C^b,c^	M: splice site (1.0) E: high impact
54	Male	4	Diplopia	Cerebrum Brainstem	No	*CCM2*	Exon 4	c.401_402insGCCC^b,c^	M: frameshift (1.0) S: frameshift (0.86)
57	Male	46	Headache	Cerebrum	Yesa	*CCM1*	Intron 13	c.1255-4_1255-2delGTA^b^	M: splice site (1.0) E: low impact
66	Female	13	Headache	Cerebrum	Yesa	*CCM1*		Same as Patient 57	
67	Male	49	No symptom	Cerebrum	Yesa	*CCM1*		Same as Patient 57	
69	Male	2	Weakness	Brainstem	No	*CCM2*	Exon 9	c.970G > A	P: 0.98, M: 0.99 S: 0.01

^a^Patients from the same family.

^b^Pathogenic variants according to American College of Medical Genetics and Genomics standards and guidelines and online predictors including PolyPhen-2 (P, HumVar score >0.5 as deleterious), MutationTaster (M, probability value of disease causing), SIFT (S, score <0.05 as deleterious in a single amino acid change; confidence score in deletion and insertion variants), and Ensembl Variant Effect Predictor (E).

^c^Novel variants found in our study.

#### Radiological finding

**Figure 2:**
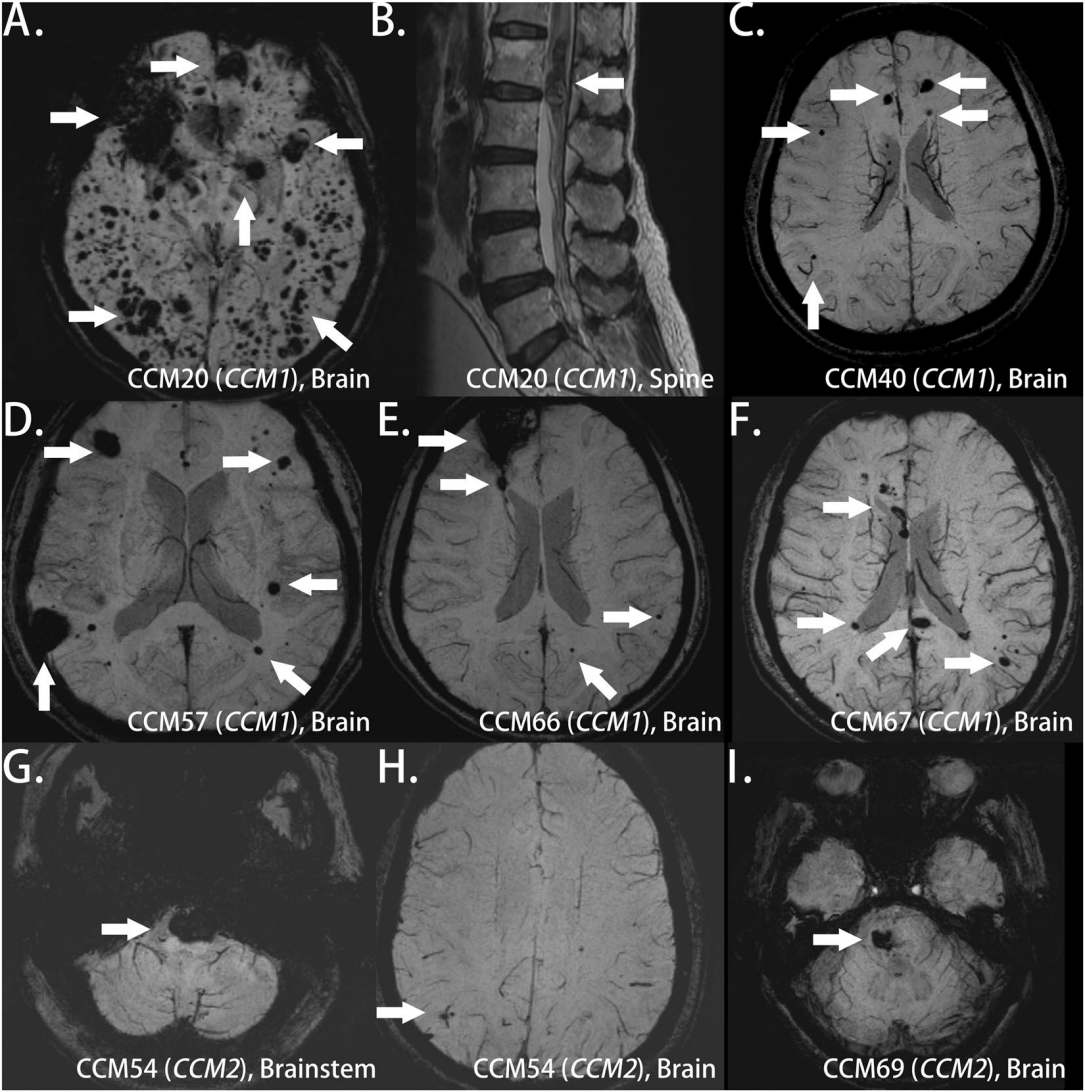
Brain MRI of patients with CCM variants. (**A**) Patient 20 with two *CCM1* variants: multiple lesions of variable sizes in hemispheres in susceptibility weighted imaging (SWI); (**B**) Patient 20 with one spinal cord lesion in a T2-weight image; (**C**) Patient 40 with one *CCM1* variant: multiple small lesions in hemispheres in SWI; (**D**–**F**) Patients 57 (**D**), 66 (**E**), and 67 (**F**) from one family with one *CCM1* variant: multiple hemisphere lesions with variable sizes in SWI; (**G**,**H**) Patient 54 with one *CCM2* variant: one prominent pontine lesion (**G**) and small lesions in bilateral hemispheres (**H**) in SWI; (**I**) Patient 69 with one *CCM2* variant of uncertain significance: one lesion in the right pons and the other in the right basal ganglion (not shown) (arrow indicates the lesion).

All the patients with pathogenic *CCM1* variants had multiple lesions in the bilateral hemisphere (Fig. 2A, C–F), and one patient had lesions in the spinal cord, as observed in the follow-up MRI study (Fig. 2B). Not only the patient (Patient 54) with one pathogenic *CCM2* variant but also the one with one *CCM2* variant of uncertain significance (Patient 69) possessed a prominent pontine lesion (Fig. 2G,H,I). Those images were reviewed by experienced radiologists based on susceptibility-weighted images^21^. The number of lesions among the six patients with pathogenic variants was not affected by genes (*p* = 0.77), sex (*p* = 0.827), or age (*p* = 0.22).

#### Variant analysis and prediction

**Figure 3:**
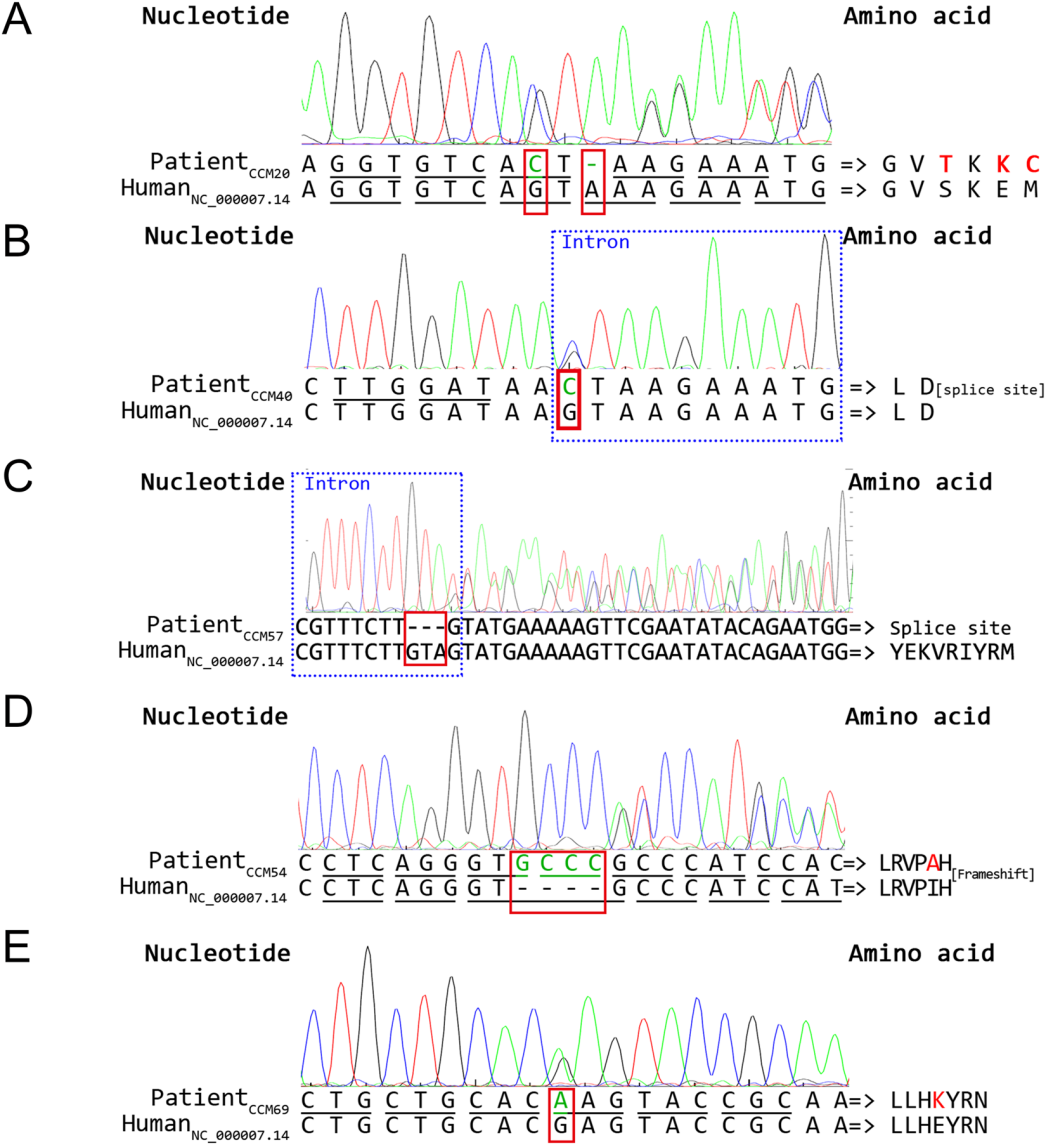
Five sequencing chromatograms of patients with variants. Each part includes a chromatogram, an interpreted sequence, reference sequences (human), and an amino acid sequence (left side). The area of intron is marked within a rectangle with a dotted line, and the sites of variants are marked within the rectangle with a solid line. The underlying lines below the sequence indicate reading frames. (**A**) *CCM1* in Patient 20: one missense (G > C) and one deletion (del-A) variant within exon 18, leading to frameshift. (**B**) *CCM1* in Patient 40: one splice site variant (G > C) at the first base pair of intron 8. (**C**) *CCM1* in Patients 57, 66, and 67: one 3-base pair deletion (del-GTA) variant in intron 13, alternating mRNA splicing. (**D**) *CCM2* in Patient 54: one 4-base pair insertion variant (ins-GCCC) within exon 4, causing frameshift. (**E**) *CCM2* in Patient 69: one missense variant (G > A) within exon 9.

**Figure 4:**
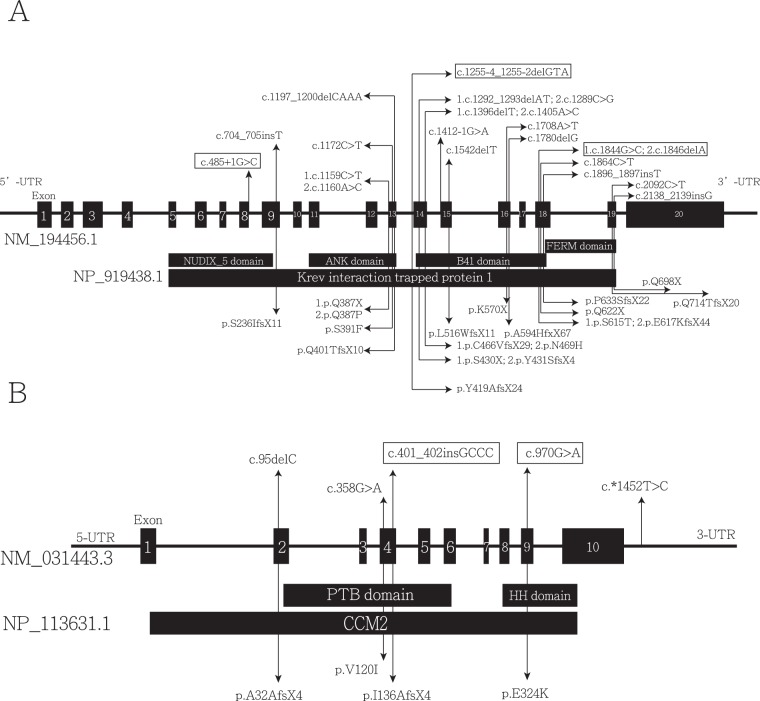
Schematic representation of the gene structure and protein domains of (**A**) *CCM1* (*KRIT1*) and (**B**) *CCM2* in a Chinese population. Each part contained an upper panel and a lower panel. The upper panel contains the genetic structure and exon position and number, and the lower panel contains a corresponding protein product and its specific domain. Each variant is marked with a double-headed arrow, indicating a variant and corresponding amino acid change, respectively. All variants reported previously in the Chinese population are shown, and variants reported in these studies are marked with rectangles.

In the variant analysis, Patient 20 had two heterozygous *CCM1* variants including one novel missense variant of uncertain significance (c.1844G > C, p.Ser615Thr) and one novel one-base pair (bp) pathogenic deletion variant (c.1846delA, p.Glu617LysfsTer44) simultaneously in the exon 18 (Fig. 3A; European Nucleotide Archive (ENA) accession number: ERZ842272). Patient 40 possessed one novel heterozygous pathogenic *CCM1* splice site variant at the beginning of intron 8 (c.485 + 1G > C) (Fig. 3B; ENA accession number: ERZ842273). Patients 57, 66, and 67 had one pathogenic *CCM1* 3-bp deletion variant (c.1255-4_1255-2delGTA or c.1255-1_1256delGTA; previously submitted ClinVar accession number: RCV000532224.2) in intron 13, leading to a splice site variant (Fig. 3C). Patient 54 possessed one novel *CCM2* pathogenic 4-bp insertion variant in exon 4 (c.401_402insGCCC, p.Ile136AlafsTer4) (Fig. 3D; ENA assessment number: ERZ842275). Finally, Patient 69 had one *CCM2* missense variant of uncertain significance in exon 9 [c.970G > A, p.Glu324Lys; minor allele frequency (MAF) of A (0.000008) in Exome Aggregation Consortium (ExAC) databank] (Fig. 3E). All previously described missense variants were located in the evolutionary conservation of sequences among functional domains (Fig. 4).

## Discussion

In familial CCM, pathogenic variants in three genes have been reported^3–5^. Our results revealed five patients with *CCM1* variants and two patients with *CCM2* variants in 41 ethnically Chinese patients with multiple CCMs or a single CCM with a relevant family history. All the patients (100%) with variants had multiple lesions. The size and number of lesions in the brain MRI were smaller and lower, respectively, in patients with *CCM2* variants than in those with *CCM1* variants. This finding is compatible with those of previous studies, indicating that *CCM2* variant carriers may have a milder phenotype than *CCM1* variant carriers^22^.

In our study, for the detection rate of variants in familial CCM (7.6% [1 of 13]; three affected members of one family among 15 patients) and sporadic (3.75% [3 of 80]; 3 sporadic cases of 80 patients) was lower than that in previous studies. Studies have reported a variable detection rate of variants ranging from 16% to 60% in sporadic cases and from 70% to 90% in familial cases^23–25^. The rate may be underestimated because DNA analysis in our study could be performed only in 41 patients (43.1%). The DNA analysis technique is essential because Sanger sequencing may miss large deletions, large insertions, or duplications. Moreover, pathogenic variants of other genes causing CCM may be another factor. A case report showed that a balanced translocation between chromosome 3 and chromosome X caused decreased zona pellucida-like domain-containing protein 1 (*ZPLD1*) expression and led to multiple cerebral cavernous hemangiomas^26^. The function of *ZPLD1* in pathogenesis of CCMs is unknown.

Among the patients with *CCM1* variants, one sporadic case (Patient 20) had a combination of a novel missense variants (c.1844G > C, p.Ser615Thr) and a novel pathogenic deletion variant (c.1846delA, p.Glu617LysfsTer44), leading to early truncation. One SNP was present at the same location with different base pair substitutions (rs780608959: c.1844G > A, p.Ser615Asn). No information was available regarding the clinical significance of this SNP in dbSNP; therefore, this missense variant was still considered to be a variant of uncertain significance. The patients with familial CCM (Patients 57, 66, and 67) had one deletion variant (c.1255-4_1255-2delGTA), which had been reported as a pathogenic splice site variant in ClinVar. One case series revealed that six affected members of one family with the same variant presented with seizure and cutaneous vascular lesions, whereas our patients only had headache or visual disturbance. The heterogeneity of clinical symptoms in patients with the same variant may relate to underlying diseases^27^, immune responses^28^, or post-transcriptional modifications^29^. Another novel splice site variant (c.485 + 1G > C), which belonged to one patient (Patient 40), affected the invariant splice donor consensus sequence, leading to abnormal splicing products.

In patients with *CCM2* variants, one patient with sporadic CCM (Patient 54) had a first reported insertion variant, leading to a frameshift and early truncation (c.401_402insGCCC, p.Ile136AlafsTer4). The other patient (Patient 69) had a *CCM2* missense variant (c.970G > A, p.Glu324Lys) of uncertain significance, which was already recorded in the ExAC databank.

In total, 14 studies have investigated *CCM1* and *CCM2* variants in ethnically Chinese populations. Detailed information is listed in Table 3 ^6–20^, and the locations of variants are shown in Fig. 4. Among those studies conducted in the ethnic Chinese population, most were case reports. One case series recruited five families and reported three novel variants causing early truncation^15^. Only two studies performed molecular screening of patients with CCMs from their brain MRI data^8,9^. These studies were conducted in 2004 and 2005, and one of the two reported only missense variants. In our study, we enrolled the largest number of patients from an ethnically Chinese population (41 patients) for molecular screening.

**Table 3:** Studies of the novel variants of *CCM1*, *CCM2*, and *CCM3* in ethnically Chinese populations.

Year	Author	Gene	Variants	Type	Peptide	Study Population
2002	Chen.	*CCM1/KRIT1*	c.2092C > T	Nonsense	p.Gln698Ter	One family
2003	Xu.	*CCM1/KRIT1*	c.1289C > G	Nonsense	p.Ser430Ter	Two families
2003	Mao.	*CCM1/KRIT1*	c.1292_1293delAT	Frameshift	p.Tyr431SerfsTer4	One family
2004	Xie.	*CCM1/KRIT1*	c.1172C > T	Missense	p.Ser391Phe	Screening
		*CCM1/KRIT1*	c.1160A > C	Missense	p.Gln387Pro	21 patients
2005	Xie.	*CCM1/KRIT1*	c.1405A > C	Missense	p.Asn469His	Screening
		*CCM1/KRIT1*	c.704_705insT	Frameshift	p.Ser236IlefsTer11	33 patients
		*CCM1/KRIT1*	c.2138_2139insG	Frameshift	p.Gln714ThrfsTer20	
		*CCM1/KRIT1*	c.1255-4G > A	Splice site	Unknown	
2006	Ji.	*CCM1/KRIT1*	c.1255-4_1255-2delGTA	Splice site	p.Tyr419AlafsTer24	One family
					p.Leu516TrpfsTer11	
2010	Lan.	*CCM1/KRIT*	c.1708A > T	Nonsense	p.Lys570Ter	One case
2011	Zhao.	*CCM1/KRIT1*	c.1197_1200delCAAA	Frameshift	p.Gln401ThrfsTer10	One family
2013	Wang.	*CCM1/KRIT1*	c.1396delT	Frameshift	p.Cys466ValfsTer29	One family
2014	Zhu.	*CCM1/KRIT1*	c.1542delT	Frameshift	p.Leu516TrpfsTer11	One family
2016	Mao.	*CCM1/KRIT1*	c.1159G > T	Nonsense	p.Gln387Ter	One family
2016	Huang.	*CCM2/MGC4607*	c.95delC	Frameshift	p.Ala32fsTer4	One family
		*CCM2/MGC4607*	c.358G > A	Missense	p.Val120Ile	
		*CCM2/MGC4607*	c.*1452T > C	Unknown	3′-UTR	
2017	Yang.	*CCM1/KRIT1*	c.1780delG	Frameshift	p.Ala594HisfsTer67	Five families
		*CCM1/KRIT1*	c.1197_1200delCAAA	Frameshift	p.Gln401ThrfsTer10	
		*CCM1/KRIT1*	c.1412-1G > A	Splice site	p.Ser471AsnfsTer2	
					p.Ser471ThrfsTer24	
2017	Yang.	*CCM1/KRIT1*	c.1864C > T	Nonsense	p.Gln622Ter	One family
2017	Wang.	*CCM1/KRIT1*	c.1896_1897insT	Frameshift	p.Pro633SerfsTer22	One family
2017	Our	*CCM1/KRIT1*	c.485 + 1G > C^a^	Splice site	—	Screening
	Study	*CCM1/KRIT1*	c.1846delA^a^	Frameshift	p.Glu617LysfsTer44	41 patients
		*CCM2/MGC4607*	c.401_402insGCCC^a^	Frameshift	p.Ile136AlafsTer4	

^a^Novel pathogenic variants according to American College of Medical Genetics and Genomics standards and guidelines.

Our study has several limitations. First, because this was a retrospective study, information bias may have been present. Second, DNA analysis was performed for less than half of our recruited patients; variants may not be detected among those without DNA analysis. Third, Sanger sequencing may fail to detect large deletions, large insertions, or duplications; therefore, other detection methods, such as multiplex ligation-dependent probe amplification (MLPA), can be applied to our recruited patients to detect more variants in future studies. Finally, there were two identified variants of uncertain significance according to ACMG standards and guidelines; therefore, biological methods such as genome editing or genotyping were necessary to verify the clinical significance of those variants and to clarify the pathogenesis of familial CCMs.

In conclusion, we identified one novel pathogenic deletion and one pathogenic splice site variant in *CCM1* and one novel pathogenic insertion variant in *CCM2*. The findings expand the knowledge related to variants present in patients with CCM, especially in the ethnic Chinese population.

## Methods

### Ethics approval and consent to participate

This study was approved by the Institutional Review Board of Chang Gung Memorial Hospital (CGMH) in Taiwan (IRB 96-1772B and 100-1666C). All examinations were performed after written informed consent was obtained. All samples were collected and analyzed in accordance with the relevant guidelines and regulations.

### Patient recruitment

We retrospectively reviewed patients who had received a diagnosis of CCM on the basis of brain MRI findings from 1998 to 2006 at CGMH. We recruited patients who had multiple lesions or a single lesion with a relevant family history. Afterward, we collected radiological and clinical assessment data including age at onset, initial presentation, underlying diseases, family history, and surgical or radiosurgical interventions.

### DNA extraction and sequencing

Venous blood samples were collected and DNA was routinely extracted using a DNA extraction kit (Stratagene La Jolla, California, United States). The extraction was monitored quantitatively and qualitatively through UV spectrophotometer absorption (ND-1000) (Nanodrop, Wilmington, United States) to verify the purity of the sample depending on the absorption at 260 nm.

Thereafter, to selectively amplify a specific DNA fragment through polymerase chain reaction (PCR), suitable primers were designed according to Primer Express version 2.0 (Applied Biosystems, United States) and online Primer3 (http://fokker.wi.mit.edu/cgi-bin/primer3/primer3_www.cgi). Our primers were short synthesized oligonucleotides, ranging from 18 to 25 bp. We used a pair of forward and reverse primers for each exon in the template DNA strand of those three genes. The primers and amplicon size of each exon are listed in Supplementary Table 1.

PCR was performed using the Fast-Run Taq Master kit (Pro Tech, Taipei, Taiwan). A typical 50-μL solution contained (1) 10 × Taq Master Mix, (2) 0.5 µM each of forward and reverse primers, (3) 100 ng of a genomic DNA template, (4) dimethyl sulfoxide for optimal performance, and (5) distilled water. The PCR reaction comprised (1) an initial denaturing step under 95 °C for 5 minutes, (2) 25–35 cycles of denaturation (95 °C, 30 seconds for each cycle), (3) primer annealing (variable, depending on the annealing temperature of the primers, 30 seconds), and (4) extension (72 °C, 30 seconds). A final extension lasted for 7 minutes at 72 °C. The final PCR products were refrigerated at 4 °C until samples were collected.

PCR products (20 ng) were purified using the Montage SEQ Sequencing Reaction Cleanup kit (Merck Millipore, Billerica, Massachusetts, United States). The resulting products were subjected to capillary electrophoresis on an ABI 3100 capillary sequencer (Applied Biosystems, United States). Sequence traces (Applied Biosystems, United States) were viewed and analyzed using the Sequencer software program.

### Sequence alignment and variant identification

We used online genomic reference sequences [*CCM1/KRIT1* (NG_012964.1/NC_000007.14), *CCM2* (NG_016295.1/NC_000007.14), and *CCM3/PDCD10* (NG_008158.1/NC_000003.12)] and mRNA transcript reference sequences [*CCM1/KRIT1* (NM_194456.1), *CCM2* (NM_031443.3), and *CCM3/PDCD10* (NM_007217.3)] for sequence alignment. Sequence alignment was performed using the online EMBOSS Water tool (http://www.ebi.ac.uk/Tools/psa/emboss_water/), and peptide analysis and translation were conducted using ExPASy (http://web.expasy.org/translate/).

The variants were considered to be novel when they were not recorded in the in PubMed, the Human Gene Mutation Database (HGMD), ExAC, gnomAD (Genome Aggregation Database), or dbSNP (https://www.ncbi.nlm.nih.gov/projects/SNP/). The clinical significance of missense or nonsense variants was predicted using SIFT (http://sift.bii.a-star.edu.sg/), PolyPhen-2 (http://genetics.bwh.harvard.edu/pph2/index.shtml), and MutationTaster (http://www.mutationtaster.org/). MutationTaster and Ensembl Variant Effect Predictor (https://asia.ensembl.org/Tools/VEP) were used to predict the clinical significance of splice site variants. The MAF of a missense variant was searched in gnomAD or ExAC databases. Variants were considered to be pathogenic according to American College of Medical Genetics and Genomics (ACMG) standards and guidelines^30^. Furthermore, we analyzed evolutionary conservation in MutationTaster. Variants in evolutionary conservation may be more inclined to have clinical significance.

We followed the Human Genome Variation Society’s (HGVS) recommendations to describe sequence variants, and we used the position of coding DNA (cDNA) sequences to exhibit the position of variants. For example, a cDNA sequence with a first nucleotide corresponded to A of ATG (translation initiation codon).

### Statistical methods and data analysis

Because of the small sample size, we used Mann–Whitney U test to compare the two groups. Correlations between two continuous variables were analyzed using Spearman rank correlation. We performed all analyses using SPSS version 24 (IBM, Armonk, NY, USA). A *p* value of < 0.05 was considered significant.

## Supplementary information

supplement 1

## References

[1] Robinson JR, Awad IA, Little JR (1991). Natural history of the cavernous angioma. J Neurosurg.

[2] Gunel M (1996). Genetic heterogeneity of inherited cerebral cavernous malformation. Neurosurgery.

[3] Notelet L (1997). Familial cavernous malformations in a large French kindred: mapping of the gene to the *CCM1* locus on chromosome 7q. J Neurol Neurosurg Psychiatry.

[4] Dupre N (2003). Linkage to the *CCM2* locus and genetic heterogeneity in familial cerebral cavernous malformation. Can J Neurol Sci.

[5] Bergametti F (2005). Mutations within the programmed cell death 10 gene cause cerebral cavernous malformations. Am J Hum Genet.

[6] Xu YL (2003). A novel Krit-1 mutation in Han family with cerebral cavernous malformation. Zhonghua Bing Li Xue Za Zhi.

[7] Mao Y (2003). Identification of a novel inheritable *CCM1* gene mutation of 671del AT in a Chinese family with cerebral cavernous malformation. Zhonghua Yi Xue Za Zhi.

[8] Xie R (2004). New mutations of the 12th exon of *CCM1* gene in Chinese patients with intracranial cavernous angiomas. Zhonghua Yi Xue Yi Chuan Xue Za Zhi.

[9] Xie R (2005). Analysis of *CCM1* gene mutations in Chinese patients with intracranial cavernous malformations. Zhonghua Yi Xue Za Zhi.

[10] Wang X (2013). Features of a Chinese family with cerebral cavernous malformation induced by a novel *CCM1* gene mutation. Chin Med J (Engl).

[11] Zhu H (2014). Familial cerebral cavernous angiomas: clinical and genetic features in a Chinese family with a frame-shift mutation in the *CCM1* gene (krit1). J Mol Neurosci.

[12] Mao CY (2016). Exome capture sequencing identifies a novel *CCM1* mutation in a Chinese family with multiple cerebral cavernous malformations. Int J Neurosci.

[13] Yang C (2017). A Novel *CCM1*/KRIT1 Heterozygous Nonsense Mutation (c.1864C>T) Associated with Familial Cerebral Cavernous Malformation: a Genetic Insight from an 8-Year Continuous Observational Study. J Mol Neurosci.

[14] Wang H (2017). A Novel KRIT1/*CCM1* Gene Insertion Mutation Associated with Cerebral Cavernous Malformations in a Chinese Family. J Mol Neurosci.

[15] Yang C (2017). Identification of a Novel Deletion Mutation (c.1780delG) and a Novel Splice-Site Mutation (c.1412-1G>A) in the CCM1/KRIT1 Gene Associated with Familial Cerebral Cavernous Malformation in the Chinese Population. J Mol Neurosci.

[16] Chen DH, Lipe HP, Qin Z, Bird TD (2002). Cerebral cavernous malformation: novel mutation in a Chinese family and evidence for heterogeneity. J Neurol Sci.

[17] Zhao Y (2011). A novel *CCM1* gene mutation causes cerebral cavernous malformation in a Chinese family. J Clin Neurosci.

[18] Ji BH (2006). A Novel Deletion Mutation in *CCM1* Gene (krit1) is Detected in a Chinese Family with Cerebral Cavernous Malformations. Yi Chuan Xue Bao.

[19] Huang WQ (2016). A Novel *CCM2* Gene Mutation Associated with Familial Cerebral Cavernous Malformation. Front Aging Neurosci.

[20] Lan MY (2010). Cavernous malformations of the central nervous system combined with cutaneous vascular lesions due to KRIT1 mutation: a case report. Clin Neurol Neurosurg.

[21] de Souza JM (2008). Susceptibility-weighted imaging for the evaluation of patients with familial cerebral cavernous malformations: a comparison with T2-weighted fast spin-echo and gradient-echo sequences. AJNR Am J Neuroradiol.

[22] Denier C (2006). Genotype-phenotype correlations in cerebral cavernous malformations patients. Ann Neurol.

[23] Stahl S (2008). Novel *CCM1*, *CCM2*, and *CCM3* mutations in patients with cerebral cavernous malformations: in-frame deletion in *CCM2* prevents formation of a *CCM1*/*CCM2*/*CCM3* protein complex. Hum Mutat.

[24] Spiegler S (2014). High mutation detection rates in cerebral cavernous malformation upon stringent inclusion criteria: one-third of probands are minors. Mol Genet Genomic Med.

[25] Merello E (2016). Genetic Screening of Pediatric Cavernous Malformations. J Mol Neurosci.

[26] Gianfrancesco F (2008). ZPLD1 gene is disrupted in a patient with balanced translocation that exhibits cerebral cavernous malformations. Neuroscience.

[27] Choquet H (2014). Association of cardiovascular risk factors with disease severity in cerebral cavernous malformation type 1 subjects with the common Hispanic mutation. Cerebrovasc Dis.

[28] Choquet H (2014). Polymorphisms in inflammatory and immune response genes associated with cerebral cavernous malformation type 1 severity. Cerebrovasc Dis.

[29] Choquet H (2016). Cytochrome P450 and matrix metalloproteinase genetic modifiers of disease severity in Cerebral Cavernous Malformation type 1. Free Radic Biol Med.

[30] Richards S (2015). Standards and guidelines for the interpretation of sequence variants: a joint consensus recommendation of the American College of Medical Genetics and Genomics and the Association for Molecular Pathology. Genet Med.

